# Metabolic effects of the schizophrenia-associated 3q29 deletion

**DOI:** 10.1038/s41398-022-01824-1

**Published:** 2022-02-17

**Authors:** Rebecca M. Pollak, Ryan H. Purcell, Timothy P. Rutkowski, Tamika Malone, Kimberly J. Pachura, Gary J. Bassell, Michael P. Epstein, Paul A. Dawson, Matthew R. Smith, Dean P. Jones, Michael E. Zwick, Stephen T. Warren, Tamara Caspary, David Weinshenker, Jennifer G. Mulle

**Affiliations:** 1 Genetics and Molecular Biology, Laney Graduate School, Emory University, Atlanta, GA 30022 USA; 2 Department of Cell Biology, School of Medicine, Emory University, Atlanta, GA 30022 USA; 3 Laboratory of Translational Cell Biology, School of Medicine, Emory University, Atlanta, GA 30022 USA; 4 Department of Human Genetics, School of Medicine, Emory University, Atlanta, GA 30022 USA; 5 Department of Pediatrics, School of Medicine, Emory University, Atlanta, GA 30022 USA; 6 Division of Pulmonary, Allergy, and Critical Care Medicine, Department of Medicine, School of Medicine, Emory University, Atlanta, GA 30022 USA; 7 Department of Genetics, School of Arts and Sciences, Rutgers University, New Brunswick, NJ USA; 8 Department of Biochemistry, School of Medicine, Emory University, Atlanta, GA 30022 USA; 9 Department of Psychiatry, Rutgers University, Piscataway, NJ 08854 USA; 10 Center for Advanced Biotechnology and Medicine, Robert Wood Johnson Medical School, Rutgers University, Piscataway, NJ 08854 USA

**Keywords:** Genetics, Neuroscience

## Abstract

The 1.6 Mb 3q29 deletion is associated with developmental and psychiatric phenotypes, including a 40-fold increased risk for schizophrenia. Reduced birth weight and a high prevalence of feeding disorders in patients suggest underlying metabolic dysregulation. We investigated 3q29 deletion-induced metabolic changes using our previously generated heterozygous B6.Del16^+/*Bdh1-Tfrc*^ mouse model. Animals were provided either standard chow (STD) or high-fat diet (HFD). Growth curves were performed on HFD mice to assess weight change (*n* = 30–50/group). Indirect calorimetry and untargeted metabolomics were performed on STD and HFD mice to evaluate metabolic phenotypes (*n* = 8–14/group). A behavioral battery was performed on STD and HFD mice to assess behavior change after the HFD challenge (*n* = 5–13/group). We found that B6.Del16^+/*Bdh1-Tfrc*^ animals preferentially use dietary lipids as an energy source. Untargeted metabolomics of liver tissue showed a strong sex-dependent effect of the 3q29 deletion on fat metabolism. A HFD partially rescued the 3q29 deletion-associated weight deficit in females, but not males. Untargeted metabolomics of liver tissue after HFD revealed persistent fat metabolism alterations in females. The HFD did not affect B6.Del16^+/*Bdh1-Tfrc*^ behavioral phenotypes, suggesting that 3q29 deletion-associated metabolic and behavioral outcomes are uncoupled. Our data suggest that dietary interventions to improve weight phenotypes in 3q29 deletion syndrome patients are unlikely to exacerbate behavioral manifestations. Our study also highlights the importance of assessing sex in metabolic studies and suggests that mechanisms underlying 3q29 deletion-associated metabolic phenotypes are sex-specific.

## Introduction

There is growing evidence that metabolic alterations can contribute to neurodevelopmental and neuropsychiatric diseases. Many inborn errors of metabolism have neurodevelopmental and neuropsychiatric manifestations [1–12]. Molecular studies of the 22q11.2 deletion have highlighted the role of oxidative stress and mitochondrial dysfunction as major contributors to synaptic phenotypes [13–15]. Mitochondrial function, oxidative stress, and small molecule dysregulation have also been implicated in the pathogenesis of idiopathic autism spectrum disorder (ASD), bipolar disorder, major depression, and schizophrenia (SZ) [16–25], highlighting some etiological similarities between syndromic and idiopathic cases of neuropsychiatric disorders.

In light of these data, we sought to investigate the link between metabolism and neurodevelopmental/psychiatric liability, using the 3q29 deletion as a model. 3q29 deletion syndrome (3q29del) is a rare (~1 in 30,000) [26, 27] genomic disorder characterized by a typically *de novo* 1.6 Mb deletion on chromosome 3 (hg19, chr3:195725000–197350000) [28–31]. The 3q29 deletion is associated with neurodevelopmental and neuropsychiatric phenotypes, including mild to moderate intellectual disability (ID) [28–31], a 19-fold increased risk for ASD [32–34], and a 20–40-fold increased risk for SZ [35–39]. Two independently generated mouse models heterozygous for the orthologous 3q29 deletion show behavioral manifestations and a reduction in body weight, suggesting a biological link between metabolic and behavioral phenotypes in 3q29del [40, 41]. The range of neurodevelopmental and neuropsychiatric manifestations in 3q29del is consistent with that observed in other copy number variant (CNV) disorders, including 22q11.2 deletion syndrome and others [42–48].

**Fig. 1:**
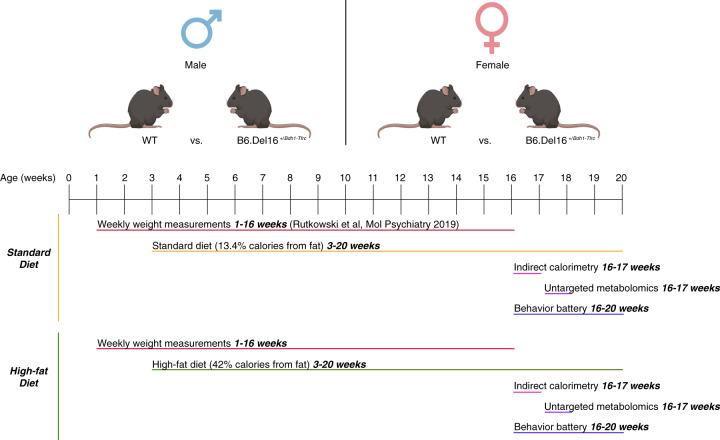
Experimental approach to interrogating the effect of the B6.Del16^+/*Bdh1-Tfrc*^ genotype on metabolism and the effect of sex on 3q29 deletion-associated metabolic phenotypes. All experiments were performed on male and female animals, and the sexes were analyzed separately. Animals were fed either standard diet chow (STD) or high-fat diet chow (HFD) from week 3 to week 20. Animals were weighed weekly from week 1 to week 16; STD weights were previously published by our group [40]. At week 16, a subset of STD- and HFD-treated animals was subjected to indirect calorimetry to assess feeding behavior and metabolic function. At the conclusion of indirect calorimetry, liver tissue was collected for untargeted metabolomics analysis. From weeks 16–20, another subset of STD- and HFD-treated animals was subjected to a behavioral battery.

The metabolic function has not been interrogated in the context of the 3q29 deletion. Existing evidence of decreased birth weight in humans with 3q29del [28], weight deficits in 3q29 deletion mouse models [40, 41], and at least four metabolic genes contained in the 3q29 interval (*Senp5*, *Tfrc*, *Bdh1*, and *Pcyt1a*) motivated our investigation of a possible unidentified metabolic disturbance associated with the 3q29 deletion (Fig. 1). Furthermore, we designed experiments to test whether metabolic disruption and adverse behavioral outcomes arise from a shared mechanism. Finally, we have specifically considered sex as a modifier of 3q29 deletion metabolic biology (Fig. 1). The new results described here have important implications for our mechanistic understanding of phenotype development in 3q29del and help to further elucidate the relationship between metabolism and neurodevelopmental and neuropsychiatric disease risk.

## Materials and methods

See Supplemental Information for detailed Methods and Protocols.

### Mouse strains and diets

All mouse work was performed under the approved guidelines of the Emory University IACUC. All studies were performed on heterozygous male and female C57BL/6N- Del16^+/*Bdh1-Tfrc*^ (B6.Del16^+/*Bdh1-Tfrc*^, MGI:6241487) mice and wild type (WT) littermates [40]. All breeding was between B6.Del16^+/*Bdh1-Tfrc*^ and WT C57BL/6 N (Charles River Laboratories). Starting at postnatal day 21, mice were fed either a standard diet (STD, LabDiet 5001) low in fat (13.4% energy from fat) or a high-fat diet (HFD, Teklad TD.88137, 42.0% energy from fat) for the remainder of their lives. No method of randomization was used to assign the diet challenge; the random assignment of mice to experimental groups (WT or B6.Del16^+/*Bdh1-Tfrc*^) was based on Mendelian inheritance. No statistical method was used to estimate sample size. Body weight was monitored weekly from 1–16 weeks of age. Indirect calorimetry and behavioral assays were performed on mice between 16–20 weeks of age. At the conclusion of indirect calorimetry, mice were euthanized, and liver tissue was collected for metabolomics analysis. An independent cohort of mice were euthanized and brain tissue was collected and immediately weighed to calculate the brain:body weight ratio. Mice were not fasted prior to euthanasia and tissue collection. The number of animals used in experiments is indicated in figure legends.

### RNA sequencing

Bulk RNAseq on liver tissue from STD-treated WT and B6.Del16^+/*Bdh1-Tfrc*^ mice were performed as previously described [49] (Fig. S1).

### Indirect calorimetry

Mouse metabolic rate was assessed by indirect calorimetry for 5 days in Oxymax chambers using the Comprehensive Lab Animal Monitoring System (Oxymax CLAMS-HC, Columbus Instruments).

### Metabolomics

Untargeted metabolomics analysis on mouse liver tissue was performed as previously described [50].

### Behavior tests

Mice were on a 12-h light/dark cycle and were given food and water *ad libitum*. Mice were 16–20 weeks of age when behavioral testing commenced. Behavioral testing was performed as previously described [40]; details on paradigms for behavioral testing can be found in Supplemental Information.

### Meta-analysis

A meta-analysis was performed using 500 randomly selected publicly available studies published since 2015 that performed metabolomics analysis on primary mouse tissue or cultured mouse cells.

### Statistical analysis

Males and females were analyzed separately in all analyses. All data is represented as mean±standard error of the mean (SEM), and the sample size and statistical tests used are included in the figure legend. Values of *p* < 0.05 were considered statistically significant. WT was set as the reference genotype and the STD was set as the reference diet for all analyses. All analyses performed in R utilized R 3.5.3 or R 4.0.4 [51]; all analyses preformed in Prism used Prism 8.3.1 (GraphPad). Growth curves, indirect calorimetry, metabolomics, RNAseq, and brain weight were analyzed in R [51]; behavioral tests were analyzed in R [51] and Prism (GraphPad).

## Results

### Reduced respiratory exchange ratio and energy expenditure, but not reduced energy consumption, in B6.Del16^+/*Bdh1-Tfrc*^ mice

**Fig. 2:**
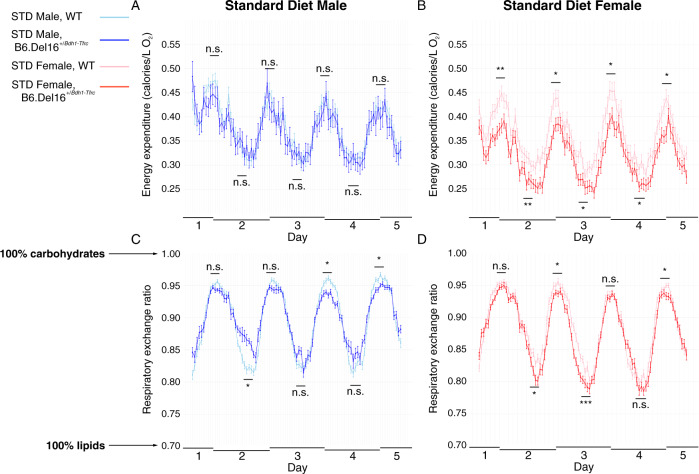
Reduced energy expenditure and respiratory exchange ratio in B6.Del16^+/*Bdh1-Tfrc*^ mice. **A** and **B** Energy expenditure for **A** male (*n* = 12 WT, 7 B6.Del16^+/*Bdh1-Tfrc*^) and **B** female (*n* = 14 WT, 12 B6.Del16^+/*Bdh1-Tfrc*^) mice on the STD over 5 days in CLAMS/Metabolic Cages. **C** and **D** RER curves for **C** male and **D** female WT and B6.Del16^+/*Bdh1-Tfrc*^ mice on the STD over 5 days in CLAMS/Metabolic Cages. Data are represented as mean ± SEM. *p < 0.05; **p < 0.01; ***p < 0.001 Statistical analysis was performed using generalized linear models.

To investigate the 3q29 deletion-associated weight deficit, we performed 5 days of indirect calorimetry on male and female B6.Del16^+/*Bdh1-Tfrc*^ mice and WT littermates. Energy expenditure was similar between WT and B6.Del16^+/*Bdh1-Tfrc*^ males (Fig. 2A); in females, energy expenditure was *reduced* in B6.Del16^+/*Bdh1-Tfrc*^ animals relative to WT (Fig. 2B). These data show that the 3q29 deletion-associated weight deficit is not due to increased energy expenditure; rather, B6.Del16^+/*Bdh1-Tfrc*^ mice burn *fewer* calories than their WT littermates. We also evaluated the respiratory exchange ratio (RER) to understand macronutrient metabolism. When lipids are used as an energy source, the RER approaches 0.7; if carbohydrates are the energy source, the RER approaches 1 [52]. The RER was reduced in both male and female B6.Del16^+/*Bdh1-Tfrc*^ mice relative to WT (Fig. 2C, D), indicating that B6.Del16^+/*Bdh1-Tfrc*^ animals preferentially use dietary lipids as an energy source as compared to WT.

Food consumption was not significantly different between male or female WT and B6.Del16^+/*Bdh1-Tfrc*^ animals after controlling for the weight (Fig. S2A). There were no differences in locomotor activity for male or female B6.Del16^+/*Bdh1-Tfrc*^ mice compared to WT littermates (Fig. S2C, D). These data show that the weight deficit in B6.Del16^+/*Bdh1-Tfrc*^ mice are not attributable to a behavioral phenotype such as decreased food consumption or increased activity levels resulting in increased energy expenditure; rather, these data support the hypothesis of altered metabolic function associated with the 3q29 deletion.

### Untargeted metabolomics reveals metabolite alterations in B6.Del16^+/*Bdh1-Tfrc*^ mice that are highly sex-dependent

**Fig. 3:**
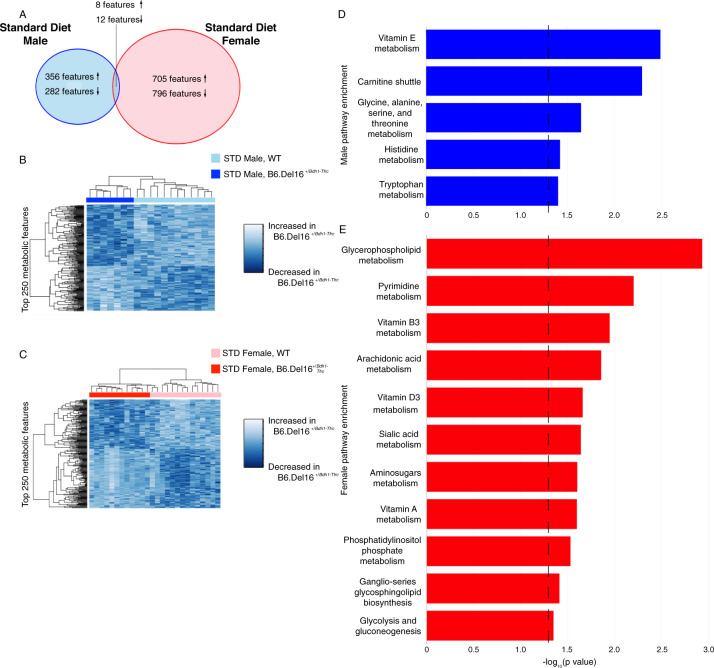
Untargeted metabolomics reveals metabolite alterations in B6.Del16^+/*Bdh1-Tfrc*^ mice that are highly sex-dependent. **A** Comparison of all nominally significant metabolomic features between the male and female datasets. Up arrows indicate metabolites significantly upregulated in B6.Del16^+/*Bdh1-Tfrc*^ samples, down arrows indicate metabolites significantly downregulated in B6.Del16^+/*Bdh1-Tfrc*^ samples. Also refer to Supplement. **B** and **C** Hierarchical clustering of **B** male (*n* = 12 WT, 7 B6.Del16^+/*Bdh1-Tfrc*^) and C) female (*n* = 14 WT, 12 B6.Del16^+/*Bdh1-Tfrc*^) samples using the top 250 ranked metabolomic features. **D** and **E** Altered pathways in B6.Del16^+/*Bdh1-Tfrc*^ mice identified via pathway enrichment analysis of **D** male and **E** female datasets. Dashed line denotes statistical significance.

To identify metabolic pathway differences, we performed untargeted metabolomic profiling on liver samples [50]. Of the nominally significant metabolic features changed between WT and B6.Del16^+/*Bdh1-Tfrc*^ samples, only 22 were shared between the male and female datasets (Fig. 3A, full details in Supplement), highlighting the sex-dependent effect of the 3q29 deletion on the metabolic environment. Using the top 250 ranked metabolic features, we were able to accurately cluster WT and B6.Del16^+/*Bdh1-Tfrc*^ samples by genotype (Fig. 3B, C). These data show that there is a substantial effect of the 3q29 deletion on the metabolic environment, and this effect is highly sex-dependent.

In addition to the lack of individual feature overlap, pathway enrichment analysis of altered features using Mummichog [53] revealed no pathway-level overlap between the sexes, again highlighting the sex-dependent effects of the 3q29 deletion on metabolism. However, pathways generally related to fat metabolism were identified in both the male and female datasets (Fig. 3D, E). Consistent with the significant reduction in RER in B6.Del16^+/*Bdh1-Tfrc*^ mice, these metabolic pathway data further demonstrate that metabolism of dietary fat may be altered in both male and female B6.Del16^+/*Bdh1-Tfrc*^ animals.

### A high-fat diet attenuates the B6.Del16^+/*Bdh1-Tfrc*^ weight deficit and affects RER in a sex-specific manner

**Fig. 4:**
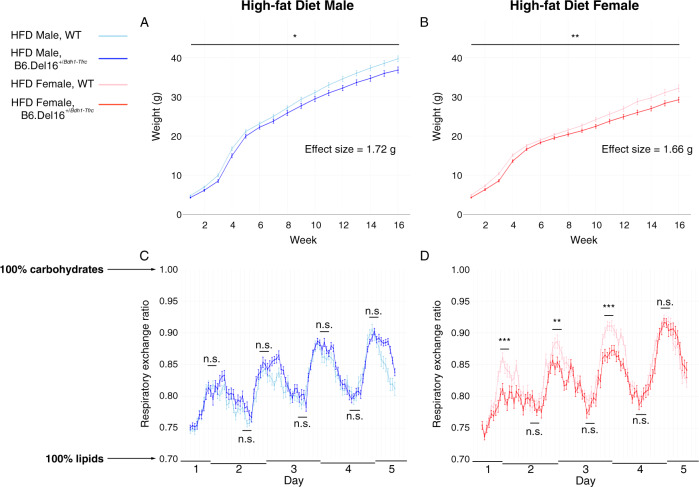
A high-fat diet reduces the B6.Del16^+/*Bdh1-Tfrc*^ weight deficit and affects RER in a sex-specific manner. **A**, **B** 16-week growth curves for HFD-treated (**A**) male (*n* = 50 WT, 30 B6.Del16^+/*Bdh1-Tfrc*^) and **B** female (*n* = 42 WT, 32 B6.Del16^+/*Bdh1-Tfrc*^) mice. **C** and **D** RER curves for **C** male (*n* = 10 WT, 10 B6.Del16^+/*Bdh1-Tfrc*^) and **D** female (*n* = 10 WT, 10 B6.Del16^+/*Bdh1-Tfrc*^) mice on the HFD over 5 days in CLAMS/Metabolic Cages. Data are represented as mean ± SEM. n.s., *p* > 0.05; *p < 0.05; **p < 0.01; ***p < 0.001 Statistical analysis of growth curves (**A**, **B**) was performed using generalized estimating equations. Statistical analysis of RER (**C**, **D**) was performed using generalized linear models.

The standard rodent chow diet (STD) contains only 13.4% of total calories from fat. Based on our finding of altered fat metabolism pathways from untargeted metabolomics, we hypothesized that increased availability of dietary fat would remedy the 3q29 deletion-associated weight deficit. We implemented a high-fat diet (HFD) challenge from postnatal day 21 to euthanasia (16–20 weeks) using a commercially available diet (TD.88137, Teklad Custom Diets, Envigo) containing 42% of total calories from fat. After HFD challenge, the weight deficit in B6.Del16^+/*Bdh1-Tfrc*^ mice was partially ameliorated in female mice but was largely unchanged in male mice (Fig. 4A, B). After the HFD challenge, male B6.Del16^+/*Bdh1-Tfrc*^ mice on the HFD weighed on average 1.72 g less than WT littermates (*p* = 0.015, Fig. 4A), compared to 1.61 g less than WT littermates on the STD [40]. Female B6.Del16^+/*Bdh1-Tfrc*^ mice on the HFD weighed on average 1.66 g less than WT littermates (*p* = 0.004, Fig. 4B), versus 2.24 g less than WT littermates on the STD [40]. When these effect sizes were considered relative to total body weight, the effect of the HFD challenge became clearer. Male B6.Del16^+/*Bdh1-Tfrc*^ mice on the HFD were 4.33% smaller than WT littermates at 16 weeks, compared to 5.16% smaller on the STD. Female B6.Del16^+/*Bdh1-Tfrc*^ mice on the HFD were 5.16% smaller than WT littermates at 16 weeks, compared to 10.29% smaller on the STD. These data demonstrate a sex-specific response to the HFD, further supporting the differential impact of the 3q29 deletion on metabolism in B6.Del16^+/*Bdh1-Tfrc*^ mice.

We also performed 5 days of indirect calorimetry on the HFD-treated B6.Del16^+/*Bdh1-Tfrc*^ and WT mice. There were no differences in RER between male WT and B6.Del16^+/*Bdh1-Tfrc*^ mice (Fig. 4C). In contrast, RER peaks remained shifted toward preferential fat use in female B6.Del16^+/*Bdh1-Tfrc*^ mice on the HFD compared to WT animals (Fig. 4D). These data suggest that the HFD rescued the shift in macronutrient utilization in males, but females may have a pronounced need for dietary lipids that was not fully satisfied by the HFD challenge. Consistent with the STD results, there were no differences between male or female WT and B6.Del16^+/*Bdh1-Tfrc*^ mice in food or water consumption (Fig. S2E, F) or activity (Fig. S2G, H). There was no difference in energy expenditure in male HFD animals (Fig. S2I) and a slight difference in energy expenditure in female HFD animals (Fig. S2J). Together, these data further support the conclusion of sex-dependent impacts of the 3q29 deletion on metabolism.

### Widespread changes in the global metabolic environment of B6.Del16^+/*Bdh1-Tfrc*^ mice after HFD challenge

**Fig. 5:**
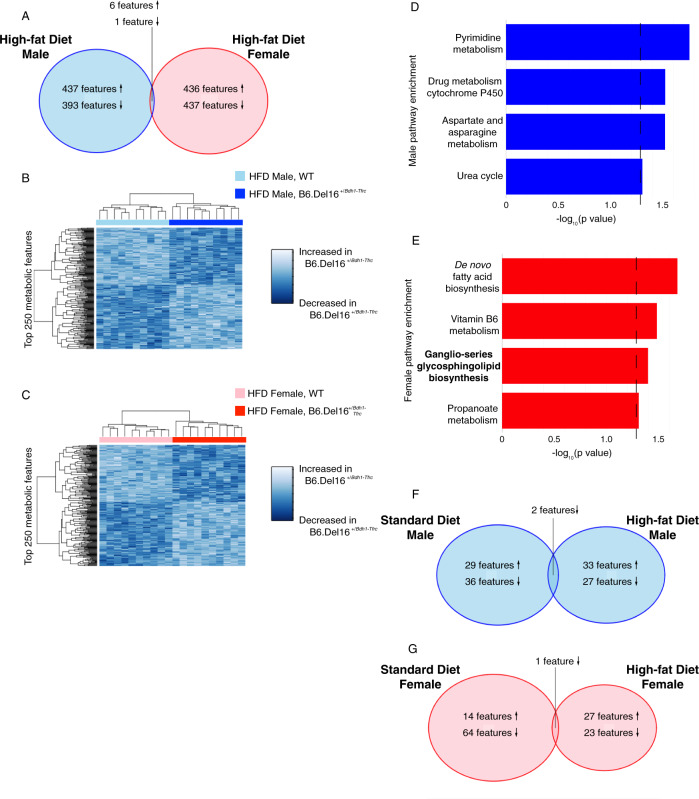
Widespread changes in the global metabolic environment of B6.Del16^+/*Bdh1-Tfrc*^ mice after HFD challenge. **A** Comparison of all nominally significant metabolomic features between the HFD-treated male and female datasets. Up arrows indicate metabolites significantly upregulated in B6.Del16^+/*Bdh1-Tfrc*^ samples, down arrows indicate metabolites significantly downregulated in B6.Del16^+/*Bdh1-Tfrc*^ samples. Also refer to Supplement. **B** and **C** Hierarchical clustering of HFD-treated (**B**) male (*n* = 10 WT, 10 B6.Del16^+/*Bdh1-Tfrc*^) and **C** female (*n* = 10 WT, 10 B6.Del16^+/*Bdh1-Tfrc*^) samples using the top 250 ranked metabolomic features. **D** and **E** Altered pathways in HFD-treated B6.Del16^+/*Bdh1-Tfrc*^ mice identified via pathway enrichment analysis of **D** male and **E** female datasets. Dashed line denotes statistical significance. Bold text denotes pathways that were identified in both the STD and HFD experiments. **F** and **G** Comparison of nominally significant annotated features between STD-treated and HFD-treated (**F**) male and **G** female datasets. Up arrows indicate metabolites significantly upregulated in B6.Del16^+/*Bdh1-Tfrc*^ samples, down arrows indicate metabolites significantly downregulated in B6.Del16^+/*Bdh1-Tfrc*^ samples. Also refer to Supplement.

We hypothesized that the HFD challenge caused changes in the global metabolic environment of B6.Del16^+/*Bdh1-Tfrc*^ animals. We performed untargeted metabolomic profiling of liver samples from HFD-treated animals [50]. Similar to the STD results, comparison of all nominally significant metabolic features between the male and female datasets revealed only 7 shared features between sexes (Fig. 5A, full details in Supplement). Using the top 250 ranked metabolites, WT and B6.Del16^+/*Bdh1-Tfrc*^ samples accurately clustered by genotype (Fig. 5B, C). Pathway enrichment analysis of altered features using Mummichog [53] identified pathways with diverse functions in both datasets (Fig. 5D, E). Pathways related to fat metabolism were identified in the female dataset, including *de novo* fatty acid biosynthesis and ganglio-series glycosphingolipid biosynthesis, as they were in the STD pathway analysis. This result is concordant with our finding of persistent RER shifts in female, but not male, B6.Del16^+/*Bdh1-Tfrc*^ mice. At the small molecule level, the HFD challenge did not restore fat metabolism functions in female B6.Del16^+/*Bdh1-Tfrc*^ mice to WT levels, supporting our hypothesis that the HFD challenge did not fully satisfy the increased metabolic demand for fat in female B6.Del16^+/*Bdh1-Tfrc*^ mice. As seen in the STD result and the lack of individual feature overlap in the HFD datasets, there was no pathway-level overlap between the sexes, further demonstrating a persistent sex effect of the 3q29 deletion on the metabolic environment after the HFD challenge.

To test for changes in metabolism resulting from the HFD challenge, we performed a direct comparison between statistically significant high-confidence annotated metabolic features from the STD and HFD cohorts, stratified by sex. In the male datasets, there were 67 and 62 significant annotated features in the STD and HFD cohorts, respectively, with only two concordant features identified in both datasets (Fig. 5F, full details in Supplement). The comparison between the female STD and HFD cohorts yielded similar results; there were 79 and 51 significant annotated features in the female STD and HFD cohorts, respectively, with only three features identified in both datasets (Fig. 5G, full details in Supplement). Further, we found that these major shifts in the metabolic environment were recapitulated at the pathway level: ganglio-series glycosphingolipid biosynthesis was the only metabolic pathway identified in both female cohorts, and there were no common pathways identified between the STD and HFD male datasets (Fig. 5E). These data demonstrate that the HFD challenge resulted in major shifts in the global metabolic environment of both male and female animals, but response to the HFD was highly sex-specific.

### HFD challenge does not affect B6.Del16^+/*Bdh1-Tfrc*^ brain size or behavioral phenotypes

Reduced brain size has been described in both mouse models of the 3q29 deletion [40, 41]. Additionally, prior work by our team identified an increased brain:body weight ratio in female, but not male, B6.Del16^+/*Bdh1-Tfrc*^ mice compared to WT littermates [40]. An increase in the brain:body weight ratio has been observed in human and animal models of starvation, lending support to the hypothesis that the brain is metabolically privileged [54–57]. Because female B6.Del16^+/*Bdh1-Tfrc*^ mice showed metabolic improvement after the HFD challenge, we hypothesized that the brain:body weight ratio in female B6.Del16^+/*Bdh1-Tfrc*^ mice on the HFD would be reduced to WT levels. We found that, consistent with prior reports, brain weight was reduced in both male (*p* = 3E-6) and female (*p* = 0.04) B6.Del16^+/*Bdh1-Tfrc*^ mice relative to WT littermates (Fig. S4A). Additionally, we found that the brain:body weight ratio in male B6.Del16^+/*Bdh1-Tfrc*^ mice was identical to that in WT animals (*p* = 1); however, the brain:body weight ratio in female B6.Del16^+/*Bdh1-Tfrc*^ mice was increased relative to WT littermates (*p* = 0.04, Fig. S4B), consistent with our previous findings in animals on the STD [40]. These data show that while the HFD challenge resulted in metabolic changes in male and female B6.Del16^+/*Bdh1-Tfrc*^ mice, and partially ameliorated the weight deficit in female B6.Del16^+/*Bdh1-Tfrc*^ mice, these positive effects did not extend to brain size or the brain:body weight ratio, indicating that early neurodevelopmental processes may not have been impacted by the HFD.

The 3q29 deletion has a well-established association with neurodevelopmental and neuropsychiatric phenotypes [28–31, 33–39, 58]; behavioral deficits have also been identified in two independent mouse models of the 3q29 deletion [40, 41]. To understand the effect of the HFD challenge on behavioral phenotypes in B6.Del16^+/*Bdh1-Tfrc*^ mice, we performed a pilot study using a battery of assays designed to test learning and memory, acoustic startle, sensorimotor gating, and amphetamine sensitivity. We replicated several phenotypes, including spatial learning and memory deficits, an elevated acoustic startle response, sensorimotor gating deficits, and attenuated amphetamine-induced locomotion [40] (full details in Supplement). We observed some sex differences between our findings and previously published results [40]; however, the present study focused on diet and was sufficiently powered for metabolic analyses but not for subtle behavioral phenotypes. There was a significant main effect of the diet challenge (*p* < 0.05) in the Morris water maze (MWM), acoustic startle, fear conditioning, and amphetamine-induced locomotor activity for both male and female B6.Del16^+/*Bdh1-Tfrc*^ mice (full details in Supplement). However, the effect of the diet was shared across genotypes; the HFD challenge did not differentially impact behavior based on genotype (full details in Supplement). Together, these data demonstrate that the HFD did not introduce any appreciable changes to behavioral phenotypes of B6.Del16^+/*Bdh1-Tfrc*^ mice, suggesting that 3q29 deletion-associated metabolic and behavioral phenotypes may arise from uncoupled, independent mechanisms.

## Discussion

This is the first study to examine changes in metabolic function associated with the 3q29 deletion. Previous work by our group and others has characterized a persistent, sex-dependent weight deficit in B6.Del16^+/*Bdh1-Tfrc*^ mice [40, 41]. In the current study, we expanded upon this work by exploring mechanisms leading to this reduced weight phenotype, with an examination of sex-dependent effects incorporated into the study design (Fig. 1). We identified pervasive sex effects of the 3q29 deletion on metabolic function. After the HFD challenge, female animals showed a change in weight, and male and female animals showed nonoverlapping changes in the metabolic profile. The HFD challenge did not rescue behavioral phenotypes in male or female animals, suggesting that 3q29 deletion-associated metabolic and behavioral phenotypes may arise from independent mechanisms. The substantial sex effects identified here show it is imperative to evaluate sex as a biological variable in metabolic studies.

In light of our data, in particular the RER shifts in STD-treated animals and the response to the HFD, we conclude that B6.Del16^+/*Bdh1-Tfrc*^ mice preferentially use lipids as an energy source. This preference for lipids was more pronounced in female animals and was partially corrected by the HFD. The HFD challenge also affected substantial changes in the global metabolic environment as assessed via untargeted metabolomics; altered metabolic pathways in B6.Del16^+/*Bdh1-Tfrc*^ mice had minimal overlap between the STD and HFD datasets. Notably, the ganglio-series glycosphingolipid biosynthesis pathway was altered in both STD- and HFD-treated female B6.Del16^+/*Bdh1-Tfrc*^ mice; this supports our conclusion that the HFD did not fully rescue fat metabolism deficits in B6.Del16^+/*Bdh1-Tfrc*^ females and further emphasizes the persistent sex-dependent effects of the 3q29 deletion.

The striking lack of overlap in male and female metabolic effects of the 3q29 deletion was unexpected. We performed a formal meta-analysis to determine whether the strong sex-dependent effects we observed in our B6.Del16^+/*Bdh1-Tfrc*^ mouse model were consistent with other metabolic studies. We randomly selected 500 metabolic studies (of 2601 that satisfied search criteria, full details in Supplement). We classified these studies according to whether both sexes were included, and whether sex-stratified analysis was conducted. Forty-four (8.8%) included both sexes, and only 17 (3.4%) analyzed the data separately by sex. Of these 17 studies, 9 (52.9%) reported substantial sex-dependent differences in the metabolome, suggesting that sex-dependent differences similar to those we observed in B6.Del16^+/*Bdh1-Tfrc*^ mice may be more pervasive than previously understood. This meta-analysis highlights the dearth of sex-stratified analyses in metabolic studies; these data highlight a pronounced knowledge gap in the field of metabolomics research and indicate there may be substantial but unappreciated sex-dependent metabolic differences in mouse models. These results have profound implications for the design of future metabolic studies; it is imperative that males and females be included and analyzed separately to rigorously assess the role of sex as a biological variable [59].

There are well-established links between sex and metabolism. Males and females have different patterns of fat deposition, and differences in fat metabolism have been identified in both humans and rodents [60–67]. Studies in rodents have revealed that the sex chromosome complement affects fat metabolism; methods such as the four core genotypes model [68] have helped to disentangle the effects of sex hormones and sex chromosomes on fat metabolism [69–72]. Gene expression studies show that a large proportion of liver-expressed transcripts in humans show sex-biased expression, and the complement of sex-biased genes are enriched for fat metabolism functions [73]. Sex hormones, specifically estrogen, also appear to have a role in sex-dependent differences in fat metabolism; oral estrogen therapy in postmenopausal women leads to well-documented changes in fat metabolism [74, 75], and endogenous levels of sex hormones also impact fat metabolism and fat distribution [60, 64, 65, 70, 76, 77]. Data from animal models have revealed pervasive roles for estrogen and estrogen-related signaling in metabolic processes including fat metabolism and storage [65]. Together with the existing literature on sex differences in fat metabolism and our finding that male and female B6.Del16^+/*Bdh1-Tfrc*^ mice are differentially affected by 3q29 deletion-associated metabolic phenotypes, this finding suggests that sex is an important consideration in defining the biological mechanisms underscoring phenotypes in 3q29del.

The links between the 3q29 deletion and metabolic function [28, 40, 41] are not unique in the broader context of recurrent CNV disorders. Weight changes and failure to thrive are associated with many recurrent CNVs, including the 22q11.2, 16p11.2, 17p11.2, and 1q21.1 loci [78–91]. Evidence has shown that pediatric feeding disorders and nutrient deficiencies can exacerbate existing neurodevelopmental and cognitive deficits [92–94]. Addressing feeding disorders and metabolic concerns in individuals with CNV disorders should be a priority, to minimize the adverse effects of poor nutrition on long-term outcomes. In the present study, we found that a HFD challenge improved metabolic phenotypes but did not affect behavioral phenotypes in B6.Del16^+/*Bdh1-Tfrc*^ mice, suggesting that a lipid-rich dietary intervention in humans with 3q29del may improve weight phenotypes and nutritional status without exacerbating behavioral phenotypes.

The effects of recurrent CNVs on growth-related phenotypes have been relatively well-described; however, the current understanding of the biological mechanisms leading to these phenotypes is lacking. Recent molecular studies have started to elucidate these mechanisms for 22q11.2 deletion syndrome; mitochondrial dysfunction has been identified as a key contributor to neuronal and synaptic defects associated with the deletion [13–15, 95]. Additionally, a recent study of a mouse model of the 16p11.2 deletion and duplication revealed mirror effects of the CNV on metabolic function [83]. However, these studies largely focused on targeted metabolic measurements, and failed to address sex as a potential mediator of metabolic phenotypes. The incorporation of untargeted approaches into studies of CNV disorders, and the rigorous interrogation of the role of sex in CNV-associated phenotypes, may expedite our understanding of the biological mechanisms at play in these complex disorders.

While previous work by our group and others has identified a significant weight deficit in mice harboring the orthologous 3q29 deletion [40, 41], this is the first study to attempt to dissect mechanisms that may be underlying this weight deficit. Reduced birth weight and failure to thrive are also commonly reported phenotypes by individuals with 3q29del and their caregivers [28], suggesting that understanding the biological mechanisms contributing to the weight deficit in our mouse model may inform our mechanistic understanding of these phenotypes in individuals with 3q29del. Our team has previously identified several neurodevelopmental phenotypes in the B6.Del16^+/*Bdh1-Tfrc*^ mouse model that are related to phenotypes reported by individuals with 3q29del, including social behavior and cognitive function deficits [28, 40]. Because pediatric feeding concerns have been shown to exacerbate existing cognitive and neurodevelopmental phenotypes [92–94], understanding if metabolic interventions could improve neurodevelopmental phenotypes in our B6.Del16^+/*Bdh1-Tfrc*^ mouse model is highly relevant and could ultimately lead to novel therapeutic strategies for individuals with 3q29del.

We found that the HFD challenge improved metabolic phenotypes but did not affect behavioral phenotypes in B6.Del16^+/*Bdh1-Tfrc*^ mice. There are several possible explanations for this outcome. First, the HFD challenge was implemented starting at postnatal day 21. Neurodevelopmental phenotypes, including ID, developmental delay (DD), ASD, are common in individuals with 3q29del. Prenatal or early postnatal mechanisms may be driving these phenotypes in the context of the 3q29 deletion. The dietary intervention in the present study may have been applied too late in development to impact these early neurodevelopmental processes. In future experiments, the HFD could be applied to pregnant or nursing dams, potentially exposing 3q29 deletion pups to abundant lipid sources earlier in development [96]. It is also possible that the behavioral assays we used and/or the sample size we evaluated could only detect large effects on behavior; the HFD may have caused subtle behavioral improvements that we were unable to detect with the behavioral battery we performed. Additionally, the HFD challenge only targeted fat metabolism, while other metabolic alterations in B6.Del16^+/*Bdh1-Tfrc*^ mice would not have been improved by the HFD. Our observation that the weight deficit was only partially ameliorated in B6.Del16^+/*Bdh1-Tfrc*^ mice supports this hypothesis, and suggests that the underlying biology of the 3q29 deletion involves multiple metabolic processes. Data from the present study suggest that metabolism and neurodevelopment may be unlinked in the context of the 3q29 deletion and may be influenced by separate sets of genes within the deletion interval.

The present study was the first to identify metabolic dysregulation in the context of the 3q29 deletion. Furthermore, we found pervasive sex-specific effects of the 3q29 deletion. These results have important implications, both for the 3q29 deletion specifically and for metabolic and mechanistic studies more generally. Our findings suggest that metabolic and behavioral phenotypes may arise from independent mechanisms in the context of the 3q29 deletion, and that these mechanisms may be sex-specific. This study underscores a critical need for metabolic and mechanistic experiments to include samples from both male and female subjects, and to analyze the data in a sex-specific manner. Due to the substantial, well-documented metabolic, medical, and neurodevelopmental and neuropsychiatric differences between males and females[60, 97–102], it is not surprising that by analyzing only one sex, or by pooling data from males and females, important metabolic insights may be obscured. Additionally, mechanistic studies in complex disorders that combine data from males and females may miss important sex-dependent differences in mechanism, which could delay advancements in available therapeutics. Together, our data highlight sex-dependent differences in metabolic function in a mouse model of the 3q29 deletion, adding to our current understanding of 3q29del and creating a framework for future mechanistic studies of complex disorders.

## Supplementary information

Supplemental Information

## Data Availability

The datasets and code supporting the current study are available from the corresponding author upon reasonable request.
