# Supplementary material for: Metabolic effects of the schizophrenia-associated 3q29 deletion
Source: Transl Psychiatry. 2022 Feb 17;12:66. doi: 10.1038/s41398-022-01824-1 (PMC8854723; doi:10.1038/s41398-022-01824-1)
Supplement: Supplementary file 1 — Supplemental Information [file 41398_2022_1824_MOESM1_ESM.docx]

**SUPPLEMENTAL MATERIALS AND METHODS**

**Animals, husbandry, and diets**

All studies were performed on male and female C57BL/6N- Del16^+/^*^Bdh1-Tfrc^* (B6.Del16^+/^*^Bdh1-Tfrc^*, MGI:6241487) mice and wild type (WT) littermates (1). All animals were maintained on a C57BL/6N background sourced from Charles River Laboratories. Mice were group housed (maximum of 5 animals per cage) during the entire experiment, except for a five-day separation during indirect calorimetry when a subset of mice were singly housed. Mice were on a 12-hour light/dark cycle and were given food and water *ad libitum*. Starting at postnatal day 21, mice were fed either a standard diet (STD, LabDiet 5001) low in fat (13.4% energy from fat) or a high-fat diet (HFD, Teklad TD.88137, 42.0% energy from fat) for the remainder of their lives. Body weight was monitored weekly from 1-16 weeks of age. Indirect calorimetry and behavioral assays were performed on mice between 16-20 weeks of age. At the conclusion of indirect calorimetry, mice were euthanized, and tissues were collected for metabolomics analysis. Mice were not fasted prior to euthanasia and tissue collection. All animal protocols were performed under the approved guidelines of the Emory University Institutional Animal Care and Use Committee. Both males and females were used in all experiments, and the data were analyzed separately. Number of animals used in experiments is indicated in figure legends.

**RNA sequencing**

Bulk RNAseq was performed on liver tissue from STD-treated WT and B6.Del16^+/^*^Bdh1-Tfrc^* mice. Liver tissue was homogenized in QIAzol (Qiagen) in a Bullet Blender Tissue Homogenizer (Next Advance, Inc.). Total RNA was isolated using the miRNeasy Mini Kit (Qiagen) with on-column DNase I treatment (Qiagen). RNA sequencing libraries were generated using the NEB Ultra II Directional kit (New England Biolabs). 50 M paired-end 150 bp read sequencing was performed on an Illumina platform. n=7 WT, 5 B6.Del16^+/^*^Bdh1-Tfrc^*; age=16-18 weeks

**Indirect calorimetry**

Mouse metabolic rate was assessed by indirect calorimetry for 5 days in Oxymax chambers using the Comprehensive Lab Animal Monitoring System (Oxymax CLAMS-HC, Columbus Instruments). Mice were singly housed with *ad libitum* access to water and food and were maintained at 20-22°C under a 12/12 hr light/dark cycle (light period 07:00-19:00). A mass-sensitive flow meter was used to maintain a constant airflow of 0.6 L/min. n=12 WT male (STD), 10 WT male (HFD), 7 B6.Del16^+/^*^Bdh1-Tfrc^* male (STD), 10 B6.Del16^+/^*^Bdh1-Tfrc^* male (HFD), 14 WT female (STD), 10 WT female (HFD), 12 B6.Del16^+/^*^Bdh1-Tfrc^* female (STD), 10 B6.Del16^+/^*^Bdh1-Tfrc^* female (HFD); age=16-18 weeks

**Metabolomics**

Untargeted metabolomics analysis on mouse liver tissue was performed as previously described (2). Briefly, supernatants were analyzed by liquid chromatography coupled to ultra-high-resolution mass spectrometry (LC-HRMS). A quality control pooled reference sample (QStd3) was included at the beginning and end of each analytical batch of 20 samples for quality control and quality assurance (2). Samples were analyzed in triplicate by liquid chromatography with Fourier transform mass spectrometry (Dionex, Ultimate 3000, Velos, Thermoe Fisher) with hydrophilic interaction liquid chromatography (HILIC) with positive electrospray ionization (ESI+) mode and reverse phase (C18) chromatography with positive electrospray ionization (ESI+) mode and resolution of 70,000 (3). Four blind replicate samples were included in the STD analysis (Figure S3A-H) and two blind replicate samples were included in the HFD analysis (Figure S3I-L) to ensure data quality. Spectral *m/z* features were acquired in scan range 85-1,250 mass-to-charge ratio (*m/z*). Raw data files were extracted using apLCMSv6.3.3 (4) with xMSanalyzer v2.0.7 (5), followed by batch correction with ComBat (6). Resulting mass spectrometry data, referred to as *m/z* features, included accurate mass *m/z*, retention time (s), and ion abundance. All samples were analyzed in triplicate, and the feature intensities were median summarized. n=12 WT male (STD), 10 WT male (HFD), 7 B6.Del16^+/^*^Bdh1-Tfrc^* male (STD), 10 B6.Del16^+/^*^Bdh1-Tfrc^* male (HFD), 14 WT female (STD), 10 WT female (HFD), 12 B6.Del16^+/^*^Bdh1-Tfrc^* female (STD), 10 B6.Del16^+/^*^Bdh1-Tfrc^* female (HFD); age=17-19 weeks

**Behavior tests**

***Morris water maze (MWM)***

The MWM was conducted to test for deficits in spatial learning and memory as previously described (7). Briefly, the MWM was conducted in a circular tank (52 inches in diameter) filled with water, made opaque with white paint, at 23°C. A hidden circular platform (30 cm in diameter) in the northwestern quadrant of the tank was present 1 cm below the surface of the water. The tank was surrounded by white walls on the north and east sides and white curtains on the west and south sides, all containing external cues for spatial reference. Mice were trained to find the hidden platform over 5 days by being released into the tank from each quadrant (north, south, east, and west) in a randomized order each day. Each trial lasted a maximum of 60 s; if a mouse did not find the platform in that time, it was guided to the platform and allowed to rest on the platform for 10 s. On the sixth day, the platform was removed from the tank and a probe trial was conducted, in which the mouse was placed in the tank at the south start point and allowed to swim for 60 s. An automated tracking system (TopScan, CleverSys) was used during training to record the latency and distance to the platform and swim speed and was used during the probe trial to record the duration and distance the mice spent in each quadrant of the maze. n=5 WT male (STD), 12 WT male (HFD), 8 B6.Del16^+/^*^Bdh1-Tfrc^* male (STD), 12 B6.Del16^+/^*^Bdh1-Tfrc^* male (HFD), 8 WT female (STD), 11 WT female (HFD), 7 B6.Del16^+/^*^Bdh1-Tfrc^* female (STD), 12 B6.Del16^+/^*^Bdh1-Tfrc^* female (HFD); age=16-20 weeks

***Acoustic startle* *and* *prepulse inhibition (PPI)***

To test for deficits in the acoustic startle response and sensorimotor gating, acoustic startle and PPI were performed as previously described (1). To test the acoustic startle response, mice were subjected to a series of increasing startle tons (75, 80, 85, 90, 95, 100, 115, and 120 db) for 40 ms each, and the response of the animal was measured by the SR-LAB startle response system (San Diego Instruments) accelerometer. A startle curve was constructed to ensure the animal was responding to the increasing stimulus. On the second day, PPI was evaluated. The mice were exposed to 6 blocks of startle conditions with each block consisting of 12 trials, so that each trial was presented to the animal 6 times. The 12 trials were randomly ordered in each block, and the animal’s response to the stimulus was measured after each trial. The 12 trials consisted of the following conditions: background (68 db) for 20 ms; startle (120 db) for 40 ms; prepulses 1-5 (PP1=70 db, PP2=74 db, PP3=78 db, PP4=82 db, PP5=86 db) for 20 ms each; and the prepulse.startle combinations (PP1.startle, PP2.startle, PP3.startle, PP4.startle, and PP5.startle), where each prepulse tone was followed by the 120 db startle tone. In the prepulse.startle trials, the mouse was exposed to the prepulse for 20 ms and the startle for 40 ms with a 100 ms gap between the two tones. Each trial was averaged over the 6 blocks, and percent PPI was calculated as:

$$\%PPI=(\frac{startle response-prepulse.startle response}{startle response})\times100$$

n=9 WT male (STD fear conditioning and PPI), 12 WT male (HFD fear conditioning and PPI), 9 B6.Del16^+/^*^Bdh1-Tfrc^* male (STD fear conditioning), 9 B6.Del16^+/^*^Bdh1-Tfrc^* male (STD PPI), 13 B6.Del16^+/^*^Bdh1-Tfrc^* male (HFD fear conditioning and PPI), 10 WT female (STD fear conditioning and PPI), 11 WT female (HFD fear conditioning and PPI), 9 B6.Del16^+/^*^Bdh1-Tfrc^* female (STD fear conditioning and PPI), 12 B6.Del16^+/^*^Bdh1-Tfrc^* female (HFD fear conditioning and PPI); age=16-20 weeks

***Fear conditioning***

To test for deficits in associative learning and memory, we performed a 3-day fear conditioning paradigm as previously described (1). Training and testing were performed in a chamber (H10-11M-TC, Coulbourn Instruments) equipped with a house light, a ceiling-mounted camera, and a speaker. On days 1 and 2, the chamber was equipped with an electric grid shock floor (H10-11M-TC-SF, Coulbourn Instruments); on day 3, the chamber was equipped with a non-shock wire mesh floor (H10-11M-TC-NSF, Coulbourn Instruments). On day 1, the animals were subjected to a 7 min training trial consisting of a 3 min acclimation period followed by three tone-shock pairings during which a tone was played for 20 s immediately followed by a 1 s, 0.5 mA foot shock. On day 2, the animals were placed back in the same chamber as day 1 and were left for 7 min without presentation of the tone or foot shock to test contextual memory. On day 3, the animals were placed in a different chamber, and the chamber floor was replaced with the non-shock wire mesh floor. The animals were in the chamber for 7 min, and the shock-associated tone was played for the last 320 s of the trial to test cued memory. Freezing behavior was automatically recorded using FreezeFrame (Coulbourn Instruments) during each trial. n=9 WT male (STD), 12 WT male (HFD), 9 B6.Del16^+/^*^Bdh1-Tfrc^* male (STD), 13 B6.Del16^+/^*^Bdh1-Tfrc^* male (HFD), 9 WT female (STD), 11 WT female (HFD), 9 B6.Del16^+/^*^Bdh1-Tfrc^* female (STD), 12 B6.Del16^+/^*^Bdh1-Tfrc^* female (HFD); age=16-20 weeks

***Amphetamine-induced locomotor activity***

To evaluate amphetamine sensitivity, amphetamine-induced locomotor activity was measured as previously described (1). The assay was performed in a locomotor chamber (San Diego Instruments) consisting of a plexiglass cage (48x25x22 cm) containing corncob bedding. The locomotor chamber was placed inside an apparatus that projected an 8x4 grid of infrared beams, with beams placed 5 cm apart. When a mouse crossed two consecutive beams, it was considered one ambulation. After a 2 hr acclimation period, mice were given an intraperitoneal injection of either saline or 7.5 mg/kg D-amphetamine and post-injection ambulations were recorded for 2 hr with the accompanying Photobeam Activity System software (San Diego Instruments). Treatments were spread over 2 weeks and were randomized, so that not all of the mice received the same injection in a given week. n=9 WT male (STD), 11 WT male (HFD), 9 B6.Del16^+/^*^Bdh1-Tfrc^* male (STD), 13 B6.Del16^+/^*^Bdh1-Tfrc^* male (HFD), 10 WT female (STD), 9 WT female (HFD), 9 B6.Del16^+/^*^Bdh1-Tfrc^* female (STD), 12 B6.Del16^+/^*^Bdh1-Tfrc^* female (HFD); age=16-20 weeks

**Meta-analysis**

A PubMed search using the keywords “mouse metabolomics” was conducted on July 7, 2020. Papers were filtered to only include publicly available studies published in English between 2015 and 2020, resulting in a list of 2601 papers. 500 papers were randomly selected using a random number generator for analysis. If a study did not use metabolomics or if a study did not use metabolomics performed on primary mouse tissue or cultured mouse cells, or if the study was not a primary research paper, it was excluded and replaced with another randomly selected study from the list of 2601 papers. Papers were reviewed and coded as one of the following to indicate how sex as a biological variable was addressed: “males only”, “females only”, “sex not specified”, “both sexes, not stratified”, or “stratified”.

**Quantification and statistical analysis**

Males and females were analyzed separately in all analyses unless otherwise specified. All data is represented as mean ± standard error of the mean (SEM) unless otherwise specified, and sample size is included in the figure legend. Values of p<0.05 were considered statistically significant. WT was set as the reference genotype and the STD was set as the reference diet for all analyses. Variance between groups was checked prior to statistical analysis. For behavior analysis, investigator was blindeds to genotype while conducting experiments; no blinding was performed for other experiments. All plots were created using the plotly R package (8) unless otherwise specified. All analyses performed in R utilized R 3.5.3 or R 4.0.4 (9). All analyses performed in Prism used Prism 8.3.1 (GraphPad). Specific details for each analysis are as follows:

***Growth curves***

Growth curve data were analyzed in R (9) using the geepack package to implement generalized estimating equations (GEE) that regressed weight measurements on genotype and age while accounting for within-subject correlation of measurements resulting from multiple time points of data collection (10-12). Age was dichotomized to “early” (1-3 weeks of age) and “late” (4-16 weeks of age) to coincide with time of weaning. All analyses were repeated after applying an inverse normal transformation to the weight data to better satisfy modeling assumptions. Results using the raw and transformed data led to identical conclusions, so the results from the analysis of the raw weight data were presented for ease of interpretation. Using the GEE framework, we performed four distinct sets of analyses. We first compared weight measurements between B6.Del16^+/^*^Bdh1-Tfrc^* and WT mice on the STD. We then compared weight measurements between an independent set of B6.Del16^+/^*^Bdh1-Tfrc^* and WT mice on the HFD. To test for sex-specific differences in effect size in each diet condition, we pooled the male and female data from that diet treatment and fit an additional GEE model that regressed weight measurements on genotype, age, sex, and a genotype-by-sex interaction term. The interaction term was tested to determine whether the effect size of the B6.Del16^+/^*^Bdh1-Tfrc^* genotype significantly differed by sex. Finally, to test for the effect of the HFD intervention on effect size within each sex, we pooled the STD and HFD data from each sex and fit a new GEE model that regressed weight measurements on genotype, age, diet, and a genotype-by-diet interaction term. The interaction term was tested to determine whether the effect size of the B6.Del16^+/^*^Bdh1-Tfrc^* genotype significantly differed between diet treatments.

***RNA sequencing***

Sequences were quality checked and aligned to the mm38 reference genome using default parameters for STAR v2.5.3 (13). Bam files were converted to sam files using Samtools (14). Gene expression was quantified using the featureCounts R package (15). The DESeq2 R package (16) was used to identify differentially expressed genes and to visualize the data.

***Indirect calorimetry***

All data were analyzed in R (9). The data were filtered to exclude observations with a respiratory exchange ratio (RER) less than 0.650 or greater than 1.05, because they are outside the dynamic range of the measurement. Observations with a negative value for cumulative food or water consumption were excluded, because they indicate periods where the animal had climbed onto the sensor. The final dataset was trimmed to remove intervals at the beginning and end of the experiment that did not have observations for every subject. Ambulations were analyzed with a simple linear model implemented through the stats R package regressing ambulations on genotype and age (9). Ambulations were averaged over all observations in the light and dark cycles for each animal, and light and dark cycle data were analyzed separately. For food and water consumption, the final value for cumulative consumption was used, and was divided by the animal’s body weight to find the g food consumed per g body weight. The relative food and water consumption data were analyzed with simple linear models implemented through the stats R package regressing relative food or water consumption on genotype and age (9). For food consumption, a mediation analysis was performed using the R package mediation to determine if body weight mediated the relationship between genotype and total food consumption (17). For energy expenditure and RER, the data were subsetted to only the peaks and troughs in the RER curve; the peak and trough were identified via manual inspection, and one interval to either side of the peak or trough was included, for a total of 3 data points per interval and 7 total intervals (the day 1 and day 5 light cycles were excluded because the entire cycle was not captured). Each interval was analyzed with a GEE implemented through the geepack R package that regressed either energy expenditure or RER on genotype and interval while accounting for within-subject correlation of measurements resulting from multiple time points of data collection (10-12).

***Metabolomics***

All data were analyzed in R (9). Median summarized ComBat batch-corrected data was used for all analyses (6). To determine the similarity between blind replicate samples, correlation tests were performed using the stats R package (9). Data filtering and feature selection with partial least squares regression and linear regression were performed using the xmsPANDA R package (18). Features missing from more than 50% of all samples, or more than 80% of samples from one group, were removed from the dataset. Stepwise feature selection was performed, where the data were first filtered based on a VIP score>1.5, and then filtered again based on a p value<0.05. All features with a p value<0.05 also had a VIP score>1.5, so the p values from linear regression were used for pathway enrichment analysis. Pathway enrichment analysis was performed using mummichog 2.3.3 with a p value<0.05 cutoff (19). For hierarchical clustering, the linear regression results from the HILIC and C18 columns were pooled, and the top 250 ranked features across the two datasets based on VIP score and p value were used as input. Hierarchical clustering was implemented via the xmsPANDA R package using Spearman correlation (18). Venn diagrams were constructed to compare male and female datasets within a diet condition, as well as to compare STD and HFD datasets within sex, using the VennDiagram R package (20). Male-female comparisons within a diet condition were performed on the top 250 ranked features. STD-HFD comparisons within sex were performed on statistically significant high-confidence annotated features, defined as an xMSannotator confidence level of 2 or 3 (21).

***Behavior tests***

*MWM*

Animals that did not swim during the probe trial were removed from all analyses. Training data (swim distance, latency to platform, and swim speed) from the STD and HFD cohorts were analyzed separately in Prism (GraphPad) using two-way repeated measures ANOVA followed by multiple comparisons with Sidak’s correction when a significant genotype effect or interaction was observed. Unpaired t-tests were implemented using the stats R package to separately analyze probe trial data (proportion of time the animals spent in the quadrant of the maze that formerly contained the platform) from the STD and HFD cohorts(9). To test for the effect of the diet intervention in the training phase, linear mixed-effects models were implemented using the lme4 R package (22). When analyzing the training phase data, we fit models with genotype as the predictor and diet and day as covariates, with subject ID as a random effect. We started with a model including up to a three-way interaction between genotype, diet, and day. To identify the most parsimonious model, we performed backward elimination via likelihood ratio tests implemented using the lmtest R package (23) and removed any higher-order interaction terms that were not significant and refit the model. We performed this process with three-way interactions followed by two-way interactions if the three-way interaction was not significant. The final models were fit with both maximum likelihood estimation and restricted maximum likelihood estimation; the fits were comparable, so the results from the models fitted with maximum likelihood are presented. P values were calculated using Satterthwaite’s method via the lmerTest R package (24). To test for the effect of the diet intervention in the probe trial, simple linear models regressing the proportion of time spent in the platform quadrant on genotype, diet, and a genotype-by-diet interaction were implemented using the stats R package (9).

*Acoustic startle*

Startle response to 70 db was excluded from the dataset for all animals. The inverse normal transformation was applied to transform the data to an approximately normal distribution. Proper transformation of the data was confirmed with the Shapiro-Wilk normality test implemented using the stats R package (9). Linear mixed-effects models were implemented using the lme4 R package (22). When analyzing the data for each diet separately, all models included genotype, decibel level, and weight as fixed effects and subject ID as a random effect. The models were fit with both maximum likelihood estimation and restricted maximum likelihood estimation; the fits were comparable, so the results from the models fitted with maximum likelihood are presented. To test for the effect of the diet intervention, we fit models with genotype as the predictor and diet, decibel level, and weight as covariates, with subject ID as a random effect. We started with a model including up to a four-way interaction between genotype, diet, decibel, and weight. To identify the most parsimonious model, we performed backward elimination via likelihood ratio tests implemented using the lmtest R package (23) and removed any higher-order interaction terms that were not significant and refit the model. We performed this process with four-way interactions, followed by three-way interactions if the four-way interaction was not significant, and followed by two-way interactions if the three-way interactions were not significant. The final models were fit with both maximum likelihood estimation and restricted maximum likelihood estimation; the fits were comparable, so the results from the models fitted with maximum likelihood are presented. P values were calculated using Satterthwaite’s method via the lmerTest R package (24).

*PPI*

Only the prepulse.startle trials were used to calculate % PPI, as shown in the equation above. The response to the PP1.startle condition (70 db prepulse) was excluded from analysis. Data from the STD and HFD cohorts were analyzed separately in Prism (GraphPad) using 2-way repeated measures ANOVA followed by multiple comparisons with Sidak’s correction when a significant genotype effect or interaction was observed. To test for the effect of the diet intervention, linear mixed-effects models were implemented using the lme4 R package (22). We fit models with genotype as the predictor and diet and prepulse decibel as covariates, with subject ID as a random effect. We started with a model including up to a three-way interaction between genotype, diet, and prepulse decibel. To identify the most parsimonious model, we performed backward elimination via likelihood ratio tests implemented using the lmtest R package (23) and removed any higher-order interaction terms that were not significant and refit the model. We performed this process with three-way interactions followed by two-way interactions if the three-way interaction was not significant. The final models were fit with both maximum likelihood estimation and restricted maximum likelihood estimation; the fits were comparable, so the results from the models fitted with maximum likelihood are presented. P values were calculated using Satterthwaite’s method via the lmerTest R package (24).

*Fear conditioning*

Data from each day of the task were analyzed separately. Data from the STD and HFD cohorts were analyzed separately in Prism (GraphPad) using 2-way repeated measures ANOVA followed by multiple comparisons with Sidak’s correction when a significant genotype effect or interaction was observed. To test for the effect of the diet intervention, linear mixed-effects models were implemented using the lme4 R package (22). All models included genotype, diet, and a genotype-by-diet interaction as fixed effects and subject ID and time as random effects. The models were fit with both maximum likelihood estimation and restricted maximum likelihood estimation; the fits were comparable, so the results from the models fitted with maximum likelihood are presented. P values were calculated using Satterthwaite’s method via the lmerTest R package (24).

*Amphetamine-induced locomotor activity*

Because the ambulation data were not normally distributed, the inverse normal function was used to transform the data to an approximately normal distribution. Proper transformation of the data as confirmed with the Shapiro-Wilk normality test implemented using the stats R package (9). Linear mixed-effects models were implemented using the lme4 R package (22). Saline was set as the reference treatment for all analyses. When analyzing the data for each diet separately, all models included genotype, treatment, and a genotype-by-treatment interaction as fixed effects and subject ID and timepoint as random effects. The models were fit with both maximum likelihood estimation and restricted maximum likelihood estimation; the fits were comparable, so the results from the models fitted with maximum likelihood are presented. To test for the effect of the diet intervention, as well as for differences in the effect of the B6.Del16^+/^*^Bdh1-Tfrc^* genotype after the HFD intervention, we fit models with genotype as the predictor and diet and treatment as covariates, with subject ID and time as random effects. We started with a model including up to a three-way interaction between genotype, diet, and treatment. To identify the most parsimonious model, we performed backward elimination via likelihood ratio tests implemented using the lmtest R package (23) and removed any higher-order interaction terms that were not significant and refit the model. We performed this process with three-way interactions followed by two-way interactions if the three-way interaction was not significant. The final models were fit with both maximum likelihood estimation and restricted maximum likelihood estimation; the fits were comparable, so the results from the models fitted with maximum likelihood are presented. P values were calculated using Satterthwaite’s method via the lmerTest R package (24).

*Brain weight*

Data were analyzed in R (9). To calculate the brain:body weight ratio, the brain weight was divided by the body weight of the animal at euthanasia. Brain weight and the brain:body weight ratio were analyzed by unpaired t-test using the stats R package (9).

**SUPPLEMENTAL RESULTS**

**Energy and water consumption are not reduced in B6.Del16^+/^*^Bdh1-Tfrc^* mice on the STD**

We performed 5 days of indirect calorimetry on male and female WT and B6.Del16^+/^*^Bdh1-Tfrc^* mice using CLAMS/Metabolic Cages (Columbus Instruments). To determine whether the weight deficit in B6.Del16^+/^*^Bdh1-Tfrc^* mice is simply due to decreased calorie intake, we evaluated food consumption. There was no significant difference in food consumption (Figure S2A) between male or female WT and B6.Del16^+/^*^Bdh1-Tfrc^* animals after controlling for the reduced weight of B6.Del16^+/^*^Bdh1-Tfrc^* animals; mediation analysis showed that including weight in the model fully accounted for the relationship between genotype and food consumption. Additionally, there were no differences between male or female WT and B6.Del16^+/^*^Bdh1-Tfrc^* mice in water consumption (Figure S2B).

**Sex-specific and diet-specific small molecule changes identified via untargeted metabolomics**

We performed untargeted metabolomics of liver samples from WT and B6.Del16^+/^*^Bdh1-Tfrc^* mice on the STD and HFD. To understand the effect of sex on the metabolic environment of animals on the STD, we compared all nominally significant features between males and females. Only 20 features were identified in both datasets (Figure 3A). Of those 20 features, 8 were enriched in samples from B6.Del16^+/^*^Bdh1-Tfrc^* animals and 12 were depleted in samples from B6.Del16^+/^*^Bdh1-Tfrc^* animals. An additional 14 features were identified in both datasets but were discordant, so they were not counted as common features. Of the 14 discordant features, 7 were enriched in samples from B6.Del16^+/^*^Bdh1-Tfrc^* males but depleted in samples from B6.Del16^+/^*^Bdh1-Tfrc^* females, while the other 7 were depleted in samples from B6.Del16^+/^*^Bdh1-Tfrc^* males but enriched in samples from B6.Del16^+/^*^Bdh1-Tfrc^* females. When we compared all nominally significant features between samples from HFD-treated males and females, a similar pattern emerged, with only 7 features identified in both datasets (Figure 5A). Of those 7 features, 6 were enriched in samples from B6.Del16^+/^*^Bdh1-Tfrc^* animals and one was depleted in samples from B6.Del16^+/^*^Bdh1-Tfrc^* animals. An additional 18 features were identified in both datasets but were discordant, so they were not counted as common features. Of the 18 discordant features, 7 were enriched in samples from B6.Del16^+/^*^Bdh1-Tfrc^* males but depleted in samples from B6.Del16^+/^*^Bdh1-Tfrc^* females, while the other 11 were depleted in samples from B6.Del16^+/^*^Bdh1-Tfrc^* males but enriched in samples from B6.Del16^+/^*^Bdh1-Tfrc^* females.

To understand the effect of the HFD on the metabolic environment, we compared the statistically significant high-confidence annotated features between STD-treated and HFD-treated males, and between STD-treated and HFD-treated females. Two features were enriched in samples from B6.Del16^+/^*^Bdh1-Tfrc^* animals in both male datasets (Figure 5F). Three additional features were identified in both datasets but were discordant, so they were not counted as common features. Of the three discordant features, one was enriched in samples from STD-treated B6.Del16^+/^*^Bdh1-Tfrc^* males but depleted in samples from HFD-treated B6.Del16^+/^*^Bdh1-Tfrc^* males, and two were depleted in samples from STD-treated B6.Del16^+/^*^Bdh1-Tfrc^* males but enriched in samples from HFD-treated B6.Del16^+/^*^Bdh1-Tfrc^* males. One feature was depleted in samples from B6.Del16^+/^*^Bdh1-Tfrc^* animals in both female datasets (Figure 5G). An additional two features were identified in both datasets but were discordant, so they were not counted as common features. Both discordant features were depleted in samples from STD-treated B6.Del16^+/^*^Bdh1-Tfrc^* females and enriched in samples from HFD-treated B6.Del16^+/^*^Bdh1-Tfrc^* females.

To evaluate the quality of the metabolomics data, we included four blind replicate samples in the STD experiment and two blind replicate samples in the HFD experiment. The STD replicate samples had an R^2^ value between 0.832 and 0.996, with a mean R^2^ of 0.963±0.055 (Figure S3A-H). The HFD replicate samples had an R^2^ value between 0.987 and 0.997, with a mean R^2^ of 0.991±0.005 (Figure S3I-L). These data demonstrate that there was good replication and consistently high data quality at the feature level across both untargeted metabolomics experiments.

**Behavioral phenotypes in B6.Del16^+/^*^Bdh1-Tfrc^* mice are not impacted by HFD treatment**

***Spatial learning and memory***

In the Morris water maze (MWM), we found no differences between male WT and B6.Del16^+/^*^Bdh1-Tfrc^* mice on the STD in swimming distance (p>0.05), latency (p>0.05), or swim speed (p>0.05) during the training portion (Figure S5A-C). Male B6.Del16^+/^*^Bdh1-Tfrc^* mice on the HFD showed increased latency (p=0.002) and swam a greater distance (p=0.002) to reach the hidden platform compared to WT littermates, but did not show any difference in swim speed (p>0.05) during the training portion (Figure S5G-I). When the data from STD- and HFD-treated males were directly compared, we observed a significant main effect of genotype on latency and swim distance (p<0.05), where male B6.Del16^+/^*^Bdh1-Tfrc^* mice took longer to reach the platform and swam a farther distance compared to WT littermates, and a significant main effect of diet on swim distance and swim speed (p<0.05), where males on the HFD swam a shorter distance and swam more slowly than males on the STD (Figure S5A-C, G-I).

Female B6.Del16^+/^*^Bdh1-Tfrc^* mice on the STD showed increased swimming distance (p=0.005), but no differences in latency (p>0.05) or swim speed (p>0.05) compared to WT littermates in the training portion of the MWM (Figure S5D-F). Female B6.Del16^+/^*^Bdh1-Tfrc^* mice on the HFD showed increased latency (p=0.025), but no differences in distance (p>0.05) or swim speed (p>0.05) compared to WT littermates in the training portion of the MWM (Figure S5J-L). When data from STD- and HFD-treated females were directly compared, we observed a significant main effect of genotype on latency and swim distance (p<0.05), where female B6.Del16^+/^*^Bdh1-Tfrc^* mice took longer to reach the platform and swam a farther distance compared to WT littermates, and a significant main effect of diet on latency, where females on the HFD took longer to reach the platform than females on the STD (Figure S5D-F, J-L).

In the probe trial of the MWM, there was no difference in the percentage of time in the quadrant that formerly contained the platform between male or female WT and B6.Del16^+/^*^Bdh1-Tfrc^* mice on the STD (Figure S5M-N). There was no difference in the percentage of time in the quadrant that formerly contained the platform between male B6.Del16^+/^*^Bdh1-Tfrc^* and WT mice on the HFD (p>0.05); however, female B6.Del16^+/^*^Bdh1-Tfrc^* mice on the HFD spent significantly less time in the platform quadrant compared to WT littermates (p=0.022, Figure S5M-N). When the data from STD- and HFD-treated males were directly compared, we found a significant main effect of diet, where males on the HFD spent more time in the quadrant that formerly contained the platform than males on the STD (p=0.02). When the data from STD- and HFD-treated females were directly compared, there were no main effects of genotype or diet (p>0.05, Figure S5M-N).

***Contextual learning and memory***

In the fear conditioning assay, male B6.Del16^+/^*^Bdh1-Tfrc^* mice on the STD showed no significant differences in freezing percentage in the training phase, but showed decreased freezing compared to WT littermates in both the context (p=0.01) and cue (p=0.004) phases (Figure S5O-Q). Likewise, male B6.Del16^+/^*^Bdh1-Tfrc^* mice on the HFD showed no significant differences in freezing percentage in the training phase, but showed decreased freezing compared to WT littermates in both the context (p=0.001) and cue (p=0.006) phases (Figure S5U-W). When the data from STD- and HFD-treated males were directly compared, we found no main effects of genotype or diet in the training phase (p>0.05, Figure S5O, U). In the context phase, there was a significant main effect of genotype, where male B6.Del16^+/^*^Bdh1-Tfrc^* mice showed decreased freezing compared to WT littermates (p=0.002, Figure S5P, V). In the tone phase, there was a significant main effect of genotype, where male B6.Del16^+/^*^Bdh1-Tfrc^* mice showed decreased freezing compared to WT littermates (p=0.001), and a significant main effect of diet, where male animals on the HFD showed increased freezing relative to males on the STD (p=0.008, Figure S5Q, W).

Female B6.Del16^+/^*^Bdh1-Tfrc^* mice on the STD had similar freezing percentages to WT littermates during all phases of the fear conditioning assay (p>0.05, Figure S5R-T). Likewise, female B6.Del16^+/^*^Bdh1-Tfrc^* mice on the HFD had similar freezing percentages to WT littermates during all phases of the task (p>0.05, Figure S5X-Z). When the data from STD- and HFD-treated females were directly compared, we found no main effects of genotype or diet in the training phase (p>0.05, Figure S5R, X). In the context phase there were no main effects of genotype or diet (p>0.05, Figure S5S, Y). In the tone phase there was a significant main effect of diet, where females on the HFD showed increased freezing relative to females on the STD (p=0.007, Figure S5T, Z).

***Acoustic startle response and sensorimotor gating***

Male and female B6.Del16^+/^*^Bdh1-Tfrc^* mice on the STD showed an increased acoustic startle response compared to WT littermates (p<0.05, FigureS5A, C). Body weight was not significantly associated with startle response for male or female animals on the STD (p>0.05), indicating that the reduced weight phenotype in B6.Del16^+/^*^Bdh1-Tfrc^* animals on the STD did not affect the measured startle response. Male and female B6.Del16^+/^*^Bdh1-Tfrc^* mice on the HFD showed an increase in acoustic startle response compared to WT littermates (p<0.05, Figure S6B, D). For male animals on the HFD, body weight was not associated with startle response (p>0.05). Weight was significantly associated with startle response in female animals on the HFD (p=0.001), suggesting that the decreased weight in female B6.Del16^+/^*^Bdh1-Tfrc^* mice on the HFD may have impacted the measured startle response. When the data from STD- and HFD-treated males were directly compared, we found a significant main effect of diet, where males on the HFD showed an increased acoustic startle response compared to males on the STD (p=0.02, Figure S6A-B). When the data from STD- and HFD-treated females were directly compared, we found a significant main effect of diet, where females on the HFD showed a decreased acoustic startle response compared to females on the STD (p=0.03), and a significant main effect of genotype, where female B6.Del16^+/^*^Bdh1-Tfrc^* mice showed a decreased acoustic startle response compared to WT littermates (p=0.02, Figure S6C-D).

Male B6.Del16^+/^*^Bdh1-Tfrc^* mice on the STD showed reduced prepulse inhibition (PPI) compared to WT littermates (p=0.02, Figure S6E), indicating a mild impairment in sensorimotor gating. Female B6.Del16^+/^*^Bdh1-Tfrc^* mice on the STD showed similar PPI to WT littermates at all prepulse levels (p>0.05, Figure S6G). Likewise, male B6.Del16^+/^*^Bdh1-Tfrc^* mice on the HFD showed significantly reduced PPI compared to WT littermates (p=0.003, Figure S6F), while female B6.Del16^+/^*^Bdh1-Tfrc^* mice on the HFD showed similar PPI to WT littermates (p>0.05, Figure S6H). When the data from STD- and HFD-treated males were directly compared, we found a significant main effect of genotype, where male B6.Del16^+/^*^Bdh1-Tfrc^* mice showed significantly reduced PPI compared to WT littermates (p=1.4E-6, Figure S6E-F). When the data from STD- and HFD-treated females were directly compared, there were no significant effects of genotype or diet (p>0.05, Figure S6G-H).

***Amphetamine sensitivity***

In the amphetamine-induced locomotor activity task, there were no differences in ambulatory activity following saline administration between male B6.Del16^+/^*^Bdh1-Tfrc^* mice on the STD and WT littermates (p>0.05). Likewise, there were no differences in ambulatory activity following saline administration between male B6.Del16^+/^*^Bdh1-Tfrc^* mice on the HFD and WT littermates (p>0.05). After administration of 7.5 mg/kg amphetamine, male B6.Del16^+/^*^Bdh1-Tfrc^* mice on the STD showed similar levels of amphetamine-induced locomotion relative to WT littermates (p>0.05, Figure S6I), whereas male B6.Del16^+/^*^Bdh1-Tfrc^* mice on the HFD showed significantly attenuated amphetamine-induced locomotion relative to WT littermates (p=0.015, Figure S6J). When the data from STD- and HFD-treated males were directly compared, we found a significant main effect of diet, where males on the HFD showed reduced activity compared to males on the STD (p=0.004), a significant genotype-by-treatment interaction, where male B6.Del16^+/^*^Bdh1-Tfrc^* mice showed reduced activity after amphetamine administration compared to WT littermates (p=0.002), and a significant diet-by-treatment interaction, where males on the HFD showed reduced activity after amphetamine administration compared to males on the STD (p=2.26E-13, Figure S6I-J).

There were no differences in ambulatory activity following saline administration between female B6.Del16^+/^*^Bdh1-Tfrc^* mice on the STD and WT littermates (p>0.05). Likewise, there were no differences in ambulatory activity following saline administration between female B6.Del16^+/^*^Bdh1-Tfrc^* mice on the HFD and WT littermates (p>0.05). After administration of 7.5 mg/kg amphetamine, female B6.Del16^+/^*^Bdh1-Tfrc^* mice on the STD showed significantly attenuated amphetamine-induced locomotion relative to WT littermates (p=9.32E-6, Figure S6K), whereas female B6.Del16^+/^*^Bdh1-Tfrc^* mice on the HFD showed similar levels of amphetamine-induced locomotion relative to WT littermates (p>0.05, Figure S6L). When the data from STD- and HFD-treated females were directly compared, we found a significant main effect of diet, where females on the HFD showed reduced activity compared to males on the STD (p=0.04), a significant genotype-by-treatment interaction, where female B6.Del16^+/^*^Bdh1-Tfrc^* mice showed reduced activity after amphetamine administration compared to WT littermates (p=2.74E-5), a significant diet-by-treatment interaction, where females on the HFD showed reduced activity after amphetamine administration compared to females on the STD (p=6.43E-13, Figure S6K-L).

**Meta-analysis of metabolomics literature**

We performed a meta-analysis of existing literature to test the hypothesis that sex is not commonly addressed as a biological variable in mouse metabolomics studies. A PubMed search for the keywords “mouse metabolomics” yielded 2,601 studies published since 2015. We randomly selected 500 studies for further analysis using a random number generator. We classified the studies based on how sex was addressed as a biological variable; studies were coded as “males only”, “females only”, “sex not specified”, “both sexes, not stratified”, or “stratified”. Of the 500 randomly selected studies, only 44 (8.8%) studied both sexes, and only 17 (3.4%) performed a stratified analysis. 69 studies used only female samples (13.8%), 248 studies used only male samples (49.6%), and 139 studies did not specify the sex of the samples (27.8%).

**SUPPLEMENTAL FIGURES**

**Figure S1. Expression of 3q29 interval genes in liver tissue from STD-treated WT and B6.Del16^+/^*^Bdh1-Tfrc^* mice.**

Expression of 3q29 interval (*Bdh1*-*Tfrc*) and flanking genes measured by bulk RNAseq in liver tissue from STD-treated WT (n=4 male, 3 female) and B6.Del16^+/^*^Bdh1-Tfrc^* (n=2 male, 3 female) mice.

n.s., p>0.05; ***, p<0.001

Statistical analysis was performed using Wald tests as implemented in the DESeq2 R package (16).

**Figure S2. Indirect calorimetry of WT and B6.Del16^+/^*^Bdh1-Tfrc^* mice on the STD and HFD. Related to Figures 2 and 4.**

A) Total food consumption for male and female WT (n=12 male, 14 female) and B6.Del16^+/^*^Bdh1-Tfrc^* (n=7 male, 12 female) mice on the STD over 5 days in CLAMS/Metabolic Cages.

B) Total water consumption for male and female WT and B6.Del16^+/^*^Bdh1-Tfrc^* mice on the STD over 5 days in CLAMS/Metabolic Cages.

C and D) Average ambulations during the light and dark cycles for C) male and D) female WT and B6.Del16^+/^*^Bdh1-Tfrc^* mice on the STD over 5 days in CLAMS/Metabolic Cages.

E) Total food consumption for male and female WT (n= 10 male, 10 female) and B6.Del16^+/^*^Bdh1-Tfrc^* (n= 10 male, 10 female) mice on the HFD over 5 days in CLAMS/Metabolic Cages.

F) Total water consumption for male and female WT and B6.Del16^+/^*^Bdh1-Tfrc^* mice on the HFD over 5 days in CLAMS/Metabolic Cages.

G and H) Average ambulations during the light and dark cycles for G) male and H) female WT and B6.Del16^+/^*^Bdh1-Tfrc^* mice on the HFD over 5 days in CLAMS/Metabolic Cages.

I and J) Energy expenditure for I) male and J) female WT and B6.Del16^+/^*^Bdh1-Tfrc^* mice on the HFD over 5 days in CLAMS/Metabolic Cages.

Data are represented as mean ± SEM. n.s., p>0.05; *, p<0.05

Statistical analysis of food consumption, water consumption, and ambulations (A-H) was performed using simple linear regression. Statistical analyses of energy expenditure (I and J) were performed using generalized linear models.

**Figure S3. Correlation between replicate samples from untargeted metabolomics.**

A, B, C, D, E, F, G, and H) Correlation between blind replicate samples for liver metabolomics of STD-treated animals using A, B, C, D) the HILIC column and E, F, G, H) the C18 column.

I, J, K, and L) Correlation between blind replicate samples for liver metabolomics of HFD-treated animals using I, J) the HILIC column and K, L) the C18 column.

**Figure S4. HFD treatment does not affect B6.Del16^+/^*^Bdh1-Tfrc^* brain size.**

A) Brain weight in HFD-treated male (n=10 WT, 10 B6.Del16^+/^*^Bdh1-Tfrc^*) and female (n=10 WT, 10 B6.Del16^+/^*^Bdh1-Tfrc^*) mice.

B) Brain weight:body weight ratio in HFD-treated male and female mice.

For each box plot, the dashed line indicates the mean value, and the solid line indicates the median. n.s., p>0.05; *, p<0.05; ***, p<0.001

Statistical analysis was performed using unpaired t-tests.

**Figure S5. Learning phenotypes in STD- and HFD-treated B6.Del16^+/^*^Bdh1-Tfrc^* mice.**

A, B, and C) MWM training A) latency to the hidden platform, B) swim distance, and C) swim speed in STD-treated males (n=5 WT, 8 B6.Del16^+/^*^Bdh1-Tfrc^*).

D, E, and F) MWM training D) latency to the hidden platform, E) swim distance, and F) swim speed in STD-treated females (n=8 WT, 7 B6.Del16^+/^*^Bdh1-Tfrc^*).

G, H, and I) MWM training G) latency to the hidden platform, H) swim distance, and I) swim speed in HFD-treated males (n=12 WT, 12 B6.Del16^+/^*^Bdh1-Tfrc^*).

J, K, and L) MWM training J) latency to the hidden platform, K) swim distance, and L) swim speed in HFD-treated females (n=11 WT, 12 B6.Del16^+/^*^Bdh1-Tfrc^*).

M and N) Percentage of time spent in the quadrant that formerly contained the platform in the probe trial of the MWM in STD- and HFD-treated M) males and N) females.

O, P, and Q) Percent freezing behavior during the fear conditioning O) training phase, P) context test, and Q) tone test in STD-treated males (n=9 WT, 9 B6.Del16^+/^*^Bdh1-Tfrc^*).

R, S, and T) Percent freezing behavior during the fear conditioning R) training phase, S) context test, and T) tone test in STD-treated females (n=9 WT, 9 B6.Del16^+/^*^Bdh1-Tfrc^*).

U, V, and W) Percent freezing behavior during the fear conditioning U) training phase, V) context test, and W) tone test in HFD-treated males (n=12 WT, 13 B6.Del16^+/^*^Bdh1-Tfrc^*).

X, Y, and Z) Percent freezing behavior during the fear conditioning X) training phase, Y) context test, and Z) tone test in HFD-treated females (n=11 WT, 12 B6.Del16^+/^*^Bdh1-Tfrc^*).

Data are represented as mean ± SEM. n.s., p>0.05; *, p<0.05; **, p<0.01; ***, p<0.001

Statistical analysis of MWM training phase and fear conditioning (A-L, O-Z) was performed using two-way repeated measures ANOVA. Statistical analysis of MWM probe trial (M and N) was performed using unpaired t-test.

**Figure S6. Acoustic startle, prepulse inhibition, and amphetamine-induced locomotion phenotypes in STD- and HFD-treated B6.Del16^+/^*^Bdh1-Tfrc^* mice.**

A, B, C, and D) Acoustic startle response in A) STD-treated males (n=9 WT, 8 B6.Del16^+/^*^Bdh1-Tfrc^*), B) HFD-treated males (n=12 WT, 13 B6.Del16^+/^*^Bdh1-Tfrc^*), C) STD-treated females (n=10 WT, 9 B6.Del16^+/^*^Bdh1-Tfrc^*), and D) HFD-treated females (n=11 WT, 12 B6.Del16^+/^*^Bdh1-Tfrc^*).

E, F, G, and H) Prepulse inhibition in E) STD-treated males (n=9 WT, 9 B6.Del16^+/^*^Bdh1-Tfrc^*), F) HFD-treated males (n=12 WT, 13 B6.Del16^+/^*^Bdh1-Tfrc^*), G) STD-treated females (n=10 WT, 9 B6.Del16^+/^*^Bdh1-Tfrc^*), and H) HFD-treated females (n=11 WT, 12 B6.Del16^+/^*^Bdh1-Tfrc^*).

I, J, K, and L) Amphetamine-induced locomotor activity in I) STD-treated males (n=9 WT, 9 B6.Del16^+/^*^Bdh1-Tfrc^*), J) HFD-treated males (n=11 WT, 13 B6.Del16^+/^*^Bdh1-Tfrc^*), K) STD-treated females (n=10 WT, 9 B6.Del16^+/^*^Bdh1-Tfrc^*), and L) HFD-treated females (n=9 WT, 12 B6.Del16^+/^*^Bdh1-Tfrc^*).

Data are represented as mean ± SEM. n.s., p>0.05; *, p<0.05; **, p<0.01; ***, p<0.001

Statistical analysis of acoustic startle and amphetamine-induced locomotor activity (A-D, I-L) was performed using linear mixed models. Statistical analysis of prepulse inhibition (E-H) was performed using two-way repeated measures ANOVA.

**REFERENCES**

1. Rutkowski TP, Purcell RH, Pollak RM, Grewenow SM, Gafford GM, Malone T, et al. Behavioral changes and growth deficits in a CRISPR engineered mouse model of the schizophrenia-associated 3q29 deletion. Mol Psychiatry. 2019.

2. Go YM, Walker DI, Liang Y, Uppal K, Soltow QA, Tran V, et al. Reference Standardization for Mass Spectrometry and High-resolution Metabolomics Applications to Exposome Research. Toxicol Sci. 2015;148(2):531-43.

3. Jones DP, Walker DI, Uppal K, Rohrbeck P, Mallon CT, Go YM. Metabolic Pathways and Networks Associated With Tobacco Use in Military Personnel. J Occup Environ Med. 2016;58(8 Suppl 1):S111-6.

4. Yu T, Park Y, Li S, Jones DP. Hybrid feature detection and information accumulation using high-resolution LC-MS metabolomics data. J Proteome Res. 2013;12(3):1419-27.

5. Uppal K, Soltow QA, Strobel FH, Pittard WS, Gernert KM, Yu T, et al. xMSanalyzer: automated pipeline for improved feature detection and downstream analysis of large-scale, non-targeted metabolomics data. BMC Bioinformatics. 2013;14:15.

6. Johnson WE, Li C, Rabinovic A. Adjusting batch effects in microarray expression data using empirical Bayes methods. Biostatistics. 2007;8(1):118-27.

7. Chalermpalanupap T, Schroeder JP, Rorabaugh JM, Liles LC, Lah JJ, Levey AI, et al. Locus Coeruleus Ablation Exacerbates Cognitive Deficits, Neuropathology, and Lethality in P301S Tau Transgenic Mice. The Journal of neuroscience : the official journal of the Society for Neuroscience. 2018;38(1):74-92.

8. Sievert C, Parmer C, Hocking T, Chamberlain S, Ram K, Corvellec M, et al. plotly: Create Interactive Web Graphics via ‘plotly.js’. R package version 460. 2017.

9. R Core Team. R: A language and environment for statistical computing. R Foundation for Statistical Computing, Vienna, Austria. 2008.

10. Halekoh U, Højsgaard S, Yan J. The R package geepack for generalized estimating equations. Journal of Statistical Software. 2006;15(2):1-11.

11. Yan J. Geepack: yet another package for generalized estimating equations. R-news. 2002;2(3):12-4.

12. Yan J, Fine J. Estimating equations for association structures. Statistics in medicine. 2004;23(6):859-74.

13. Dobin A, Davis CA, Schlesinger F, Drenkow J, Zaleski C, Jha S, et al. STAR: ultrafast universal RNA-seq aligner. Bioinformatics. 2013;29(1):15-21.

14. Danecek P, Bonfield JK, Liddle J, Marshall J, Ohan V, Pollard MO, et al. Twelve years of SAMtools and BCFtools. GigaScience. 2021;10(2).

15. Liao Y, Smyth GK, Shi W. featureCounts: an efficient general purpose program for assigning sequence reads to genomic features. Bioinformatics. 2014;30(7):923-30.

16. Love MI, Huber W, Anders S. Moderated estimation of fold change and dispersion for RNA-seq data with DESeq2. Genome Biol. 2014;15(12):550.

17. Tingley D, Yamamoto T, Hirose K, Keele L, Imai K. Mediation: R package for causal mediation analysis. 2014.

18. Uppal K. xmsPANDA: Predictive And Network Discovery Analysis (xmsPANDA): R package for biomarker discovery and biomarker-driven network analysis for studying disease mechanisms using metabolomics. R package version 1.0.7.5. 2018.

19. Li S, Park Y, Duraisingham S, Strobel FH, Khan N, Soltow QA, et al. Predicting network activity from high throughput metabolomics. PLoS Comput Biol. 2013;9(7):e1003123.

20. Chen H. VennDiagram: generate high-resolution venn and euler plots. R package version 1620. 2018.

21. Uppal K, Walker DI, Jones DP. xMSannotator: an R package for network-based annotation of high-resolution metabolomics data. Analytical chemistry. 2017;89(2):1063-7.

22. Bates D, Mächler M, Bolker B, Walker S. Fitting linear mixed-effects models using lme4. arXiv preprint arXiv:14065823. 2014.

23. Zeileis A, Hothorn T. Diagnostic checking in regression relationships. 2002.

24. Kuznetsova A, Brockhoff PB, Christensen RHB. lmerTest package: tests in linear mixed effects models. Journal of statistical software. 2017;82(13).
